# Morpho-physiological integrators, transcriptome and coexpression network analyses signify the novel molecular signatures associated with axillary bud in chrysanthemum

**DOI:** 10.1186/s12870-020-02336-0

**Published:** 2020-04-07

**Authors:** Sagheer Ahmad, Cunquan Yuan, Qingqing Yang, Yujie Yang, Tangren Cheng, Jia Wang, Huitang Pan, Qixiang Zhang

**Affiliations:** 1 Beijing Key Laboratory of Ornamental Plants Germplasm Innovation & Molecular Breeding, National Engineering Research Center for Floriculture, Beijing Laboratory of Urban and Rural Ecological Environment, Key Laboratory of Genetics and Breeding in Forest Trees and Ornamental Plants of Ministry of Education, School of Landscape Architecture, Beijing Forestry University, Beijing, 100083 China; 2 Beijing Advanced Innovation Center for Tree Breeding by Molecular Design, Beijing Forestry University, Beijing, 100083 China

**Keywords:** High temperature, Axillary bud, WGCNA, Chrysanthemum

## Abstract

**Background:**

Axillary bud is an important agronomic and economic trait in cut chrysanthemum. Bud outgrowth is an intricate process controlled by complex molecular regulatory networks, physio-chemical integrators and environmental stimuli. Temperature is one of the key regulators of bud’s fate. However, little is known about the temperature-mediated control of axillary bud at molecular levels in chrysanthemum. A comprehensive study was designed to study the bud outgrowth at normal and elevated temperature in cut chrysanthemum. Leaf morphology, histology, physiological parameters were studied to correlate the leaf activity with bud morphology, sucrose and hormonal regulation and the molecular controllers.

**Results:**

Temperature caused differential bud outgrowth along bud positions. Photosynthetic leaf area, physiological indicators and sucrose utilization were changed considerable due to high temperature. Comparative transcriptome analysis identified a significant proportion of bud position-specific genes.Weighted Gene Co-expression Network Analysis (WGCNA) showed that axillary bud control can be delineated by modules of coexpressed genes; especially, MEtan3, MEgreen2 and MEantiquewhite presented group of genes specific to bud length. A comparative analysis between different bud positions in two temperatures revealed the morpho-physiological traits associated with specific modules. Moreover, the transcriptional regulatory networks were configured to identify key determinants of bud outgrowth. Cell division, organogenesis, accumulation of storage compounds and metabolic changes were prominent during the bud emergence.

**Conclusions:**

RNA-seq data coupled with morpho-physiological integrators from three bud positions at two temperature regimes brings a robust source to understand bud outgrowth status influenced by high temperature in cut chrysanthemum. Our results provide helpful information for elucidating the regulatory mechanism of temperature on axillary bud growth in chrysanthemum.

## Background

Branches, besides being the architectural determinants, provide plants with plenty of shapes and ornamental values. Axillary bud is one of the most important agronomic traits related to cut floral beauty; especially in chrysanthemum where excessive outgrowth of axillary branches is a major drawback in market success of cut flowers. Since antiquity, strides have been directed towards the temperature control, manual disbudding and the molecular regulation of axillary growth. However, due to limited access to the complex genomic structure of this crop, the bud control remained a mystery for the researchers. Recently, the whole genome sequencing [1] have facilitated the tracing of axillary bud regulation at molecular levels. With this breakthrough, a comprehensive assessment of molecular regulatory mechanism of bud outgrowth controlled by temperature can point out important thermal inhibitors of axillary bud.

High-definition transcriptomic investigations in different plants have provided valuable insights into the molecular pathways and networks, and their interactions with different aspects of bud outgrowth [2–7]. The molecular mechanism behind bud outgrowth has been demonstrated to some depth in some plants [2, 8]. Carbohydrate distribution, photoassimilate supply and the accumulation rate of storage compounds during bud initiation are important regulators of bud outgrowth [9–11]. Moreover, epigenetic imprinting and hormonal signal transduction have also been considered as pivotal regulators of bud kinetics [12–17]. Along with these regulators, temperature is also considered pivotal when studying bud opening and outgrowth.

However, after the report of Faust and Heins [18], stating that chrysanthemum (Powerhouse) produces little axillary shoots at 35 °C; no further progress could be made in chrysanthemum. Temperature elevation beyond a certain level can cause sterile spikelet [19]; as suggested to be due to high temperature [20–23]. The recent researches have shown many spikelet-related DEGs sensitive to heat stress [19] and a high temperature exposure of 39 °C suppressed the spikelet fertility [23]. Gene expression profiling of rice panicles growing at 40 °C identified DEGs involving mainly in transport, transcriptional regulation, stress response and cellular homeostasis [24]. Moreover, the pattern of gene expression due to heat stress and the regulation model based on ROS (reactive oxygen species) along with transcriptome implied the importance of ROS balance in panicle growth [24]. According to a report on rice genome, one half of the genes (50.4%) expressed at 25 °C and another half (50.2%) expressed at 30 °C; moreover, temperature stimulated many transcription factor families, including WRKY, bZIP, and MYB [25]. Thus far, nothing is documented on the transcriptional mechanism of axillary bud outgrowth in response to high temperature in cut chrysanthemum.

Temperature extremes can cause water stress, thereby triggering the reactive oxygen species (ROS) [26–28]. Excessive accumulation of ROS can cause oxidative harm to lipids leading to the production of MDA (malondialdehyde), an indicator of oxidative stress level in plants [29]. Plants use antioxidant defense system to cope with the accumulation of ROS. This system uses water-soluble reducing agents (e.g., glutathione and ascorbate), lipid-soluble antioxidants (e.g., carotene and α-tocopherol) and enzymes like SOD (superoxide dismutase) [27, 30]. Thus, deciphering the accumulation of SOD and MDA can expose the damages caused by water stress and/or temperature stress. SOD triggers the catalysis of superoxide radical to H_2_O_2_ and O_2_ through a spontaneous and extremely fast reaction in order to defend the plant cells from the products of superoxide radical reaction. The potentially toxic compound is reduced to water through the action of enzymes, such as catalase (CAT) and peroxidase (POD) [31, 32]. CAT converts hydrogen per oxide (H_2_O_2_) to H_2_O and O_2_.

Chrysanthemum is the second most important floriculture crop in worldwide floriculture trade [33, 34], sharing 30% of the total cut flower production in the world. Axillary branching is a vital end-user quality attribute of cut chrysanthemum. Diminished axillary bud growth is highly desirable to provide high market price for cut flowers. The accessibility to its genomic sequences and the transcriptome [1] along with RNA-Sequencing can do a great deal to reveal genetic regulation of bud initiation and outgrowth in cut chrysanthemum. A few efforts have been made to understand the transcriptomic basis of bud development [2, 3, 6]. However, no such strides have been made to find the temperature-mediated molecular mechanism behind bud initiation and outgrowth in chrysanthemum.

To the best of our knowledge, a comparative transcriptome analysis of bud outgrowth, at different positions, at different temperatures has not yet been performed in cut chrysanthemum. Here, we applied RNA-Seq technology to analyze axillary bud transcriptome at two different temperatures (i.e., 25 °C and 35 °C). The data were dissected to ascertain transcriptome dynamics and the transcriptional regulatory networks linked with bud outgrowth. The coexpressed gene modules were identified for each bud position under contrasting temperatures. Moreover, spacial control of photoassimilates and the chemical homeostasis by leaves were also studied as indirect controlling mechanism for bud regulation. Thus, this study gives valuable insights into the molecular regulatory mechanism and the pivotal factors governing axillary bud outgrowth.

## Results

High temperature causes morpho-physiological changes in plants [35] and removing leaves retards bud outgrowth, showing the importance of leaf-derived physio-metabolic factors in axillary bud control [36]. Therefore, connecting leaf dynamics with bud outgrowth can be an effective tool to understand the leaf-mediated influence of temperature.

### Bud positions express differently for high and normal temperature

At an extended plant height (35–40 cm) the plant shows maximum bearing of axillary buds at all positions (Supplementary Fig. 1). At this stage, high density of axillary growth was seen in normal temperature regime as compared to high temperature. Top buds retained the growth potential at both the temperatures. However, high temperature almost completely checked the outgrowth of top axillary buds (TAB). More axillary growth was seen at lower axillary positions at 25 °C, whereas it was restricted in high temperature regime, suggesting that different bud positions respond differently against temperature changes.

### Temperature indirectly affects bud kinetics via leaf

**Fig. 1:**
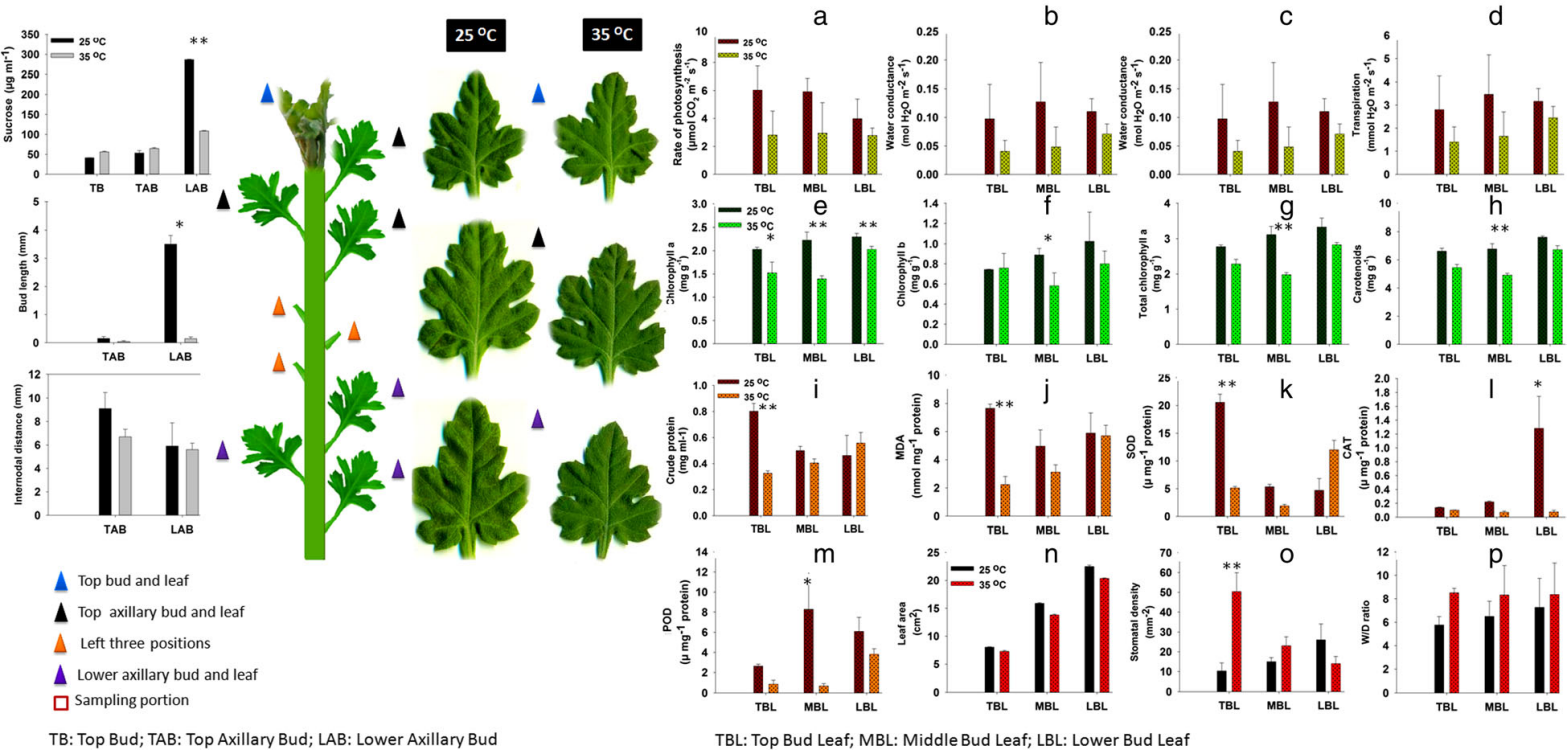
Bud length, sucrose concentration and internodal distance (extreme left). Sampling plan for axillary buds and leaves of *Chrysanthemum morifolium* ‘Jinba’ under contrasting temperatures (left) and three leaf positions used for analysis (middle) and the leaf parameters (right). The leaf characteristics include, (**a**) photosynthesis, (**b**) water conductance, (**c**) intercellular CO_2_ concentration, (**d**) transpiration, (**e**) chlorophyll a, (**f**) chlorophyll b, (**g**) total chlorophyll, (**h**) carotenoids, (**i**) crude protein, (**j**) MDA, (**k**) SOD, (**l**) CAT, (**m**) POD, (**n**) leaf area, (**o**) stomatal density and (**p**) W/D ratio. Data are shown as mean ± SE of three biological replicates. Asterisks on specific terms show significant differences between the treatment conditions 25 °C and 35 °C for each bud position at *p* < 0.05(*) and *p* < 0.01(**) level

Among the leaf morphological indices, leaf area (Fig. 1o), wet to dry mass ratio (Fig. 1q) and stomatal density (Fig. 1p) were observed in 11 days post-transplantation in contrasting temperature regimes. Leaf area was marginally high in normal temperature plants as compared to high temperature. High wet to dry mass ratio was seen in high temperature plants, suggesting more accumulation of photoassimilates in the leaf. However, the stomatal density was significantly high in top bud leaves (50.33 mm^− 2^, *p* < 0.01) in 35 °C leaves as compared to 24 °C where only 10.33 stomata were found in 1 mm^− 2^ surface area.

More photosynthesis was observed in 25 °C plants as compared to 35 °C (Fig. 1a). Same was the case with transpiration (Fig. 1d) where normal temperature caused more gas exchange. In the case of water conductance (Fig. 1b), considerable difference was observed at different temperatures as compared to intercellular CO_2_ concentration (Fig. 1c). Significant changes occurred in chlorophyll contents (Fig. 1e-g) at different temperatures, suggesting positive correlation between photosynthetic pigments and normal temperature. Carotenoids were also observed to be more fluctuating at upper bud leaves due to temperature change (Fig. 1h).

### Physiological responses of leaf against temperature

A significant difference (*p* < 0.01) was observed in the top bud leaves (TBL) in crude protein concentration (Fig. 1i). 25 °C leaves showed significant high MDA content in top bud leaves (Fig. 1j) whereas negligible differences were seen at other bud positions. In case of SOD concentration (Fig. 1k), top leaves showed high concentration in normal temperature and lower bud leaves (LBL) showed high concentration in high temperature. However, the difference was more significant in TBL as compared to LBL. For POD (Fig. 1m), the maximum concentration was shown by TBL in normal temperature than high temperature. However, the TBL and LBL did not show significant differences, except that the POD concentration was little high in normal temperature leaves at both leaf positions. The catalase activity was significant at LBL and CAT concentration was the maximum at 25 °C than 35 °C. However, at other leaf positions, there was no considerable difference between the leaves at contrasting temperatures (Fig. 1l).

### Ultra-structural leaf and bud attributes as influenced by temperature variation

**Fig. 2:**
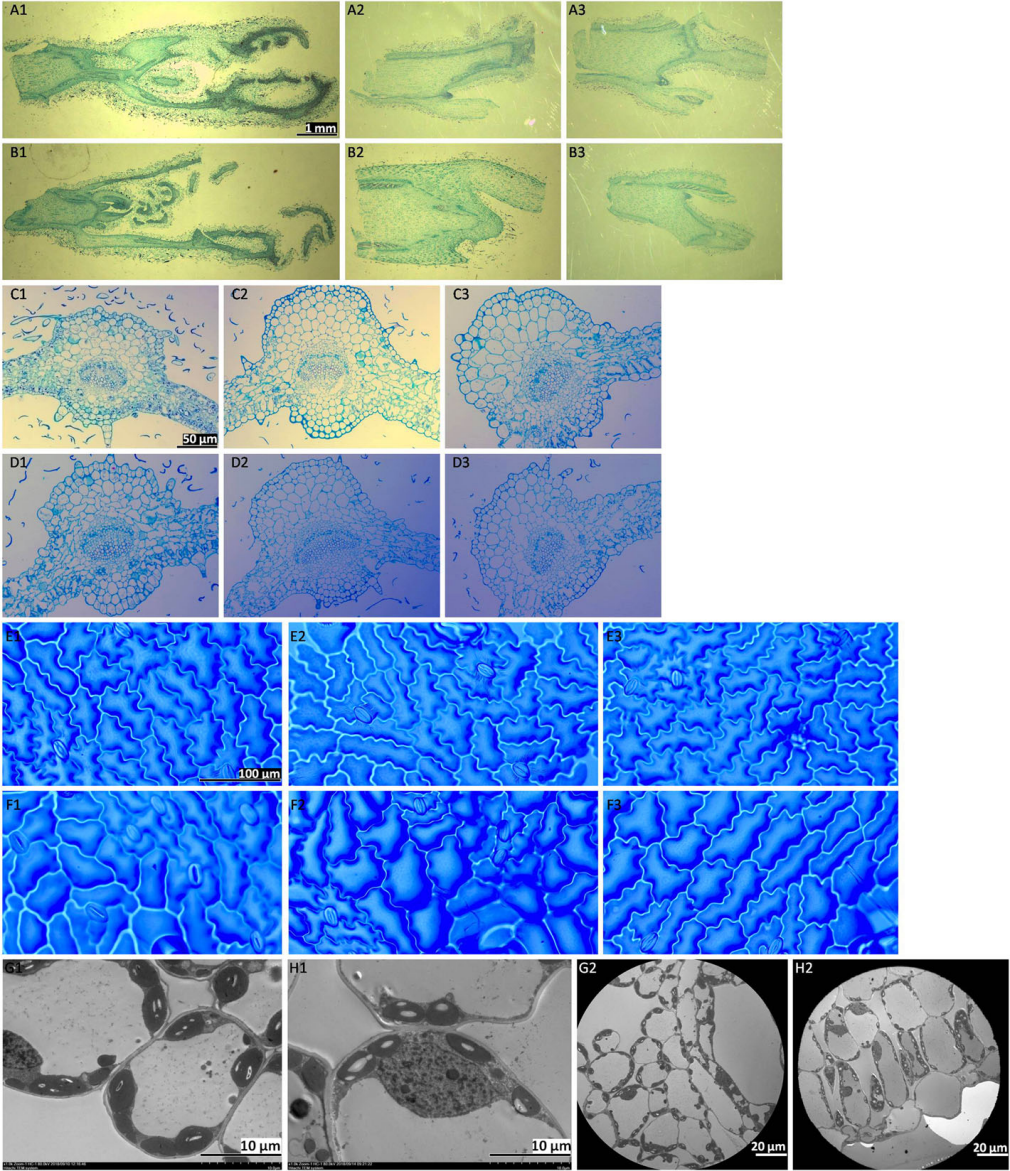
Microscopic examinations of bud (A,B) and leaf (C-H) characteristics under two different temperatures. Paraffin sectioning pictures of bud at 25 °C (TB:A1, TAB:A2; LAB:A3) and 35 °C (TB:B1, TAB:B2; LAB:B3). Paraffin sectioning pictures of leaf cross section at 25 °C (TBL:C1, MBL:C2; LBL:C3) and 35 °C (TBL:D1, MBL:D2; LBL:D3). Leaf outer surface pictures at 25 °C (TBL:E1, MBL:E2; LBL:E3) and 35 °C (TBL:F1, MBL:F2; LBL:F3). Transmission electron micrographs at 25 oC (TBL: G1, G2) and 35 °C (TBL: H1, H2)

Paraffin sectioning buds showed that high temperature prohibited the bud outgrowth to some extent in all bud positions (Fig. 2a, b). Top buds showed a little restricted growth in high temperature as compared to normal temperature (Fig. 2; A1, B1). However, the marked difference was noted at top axillary buds where bud outgrowth was completely checked by high temperature at the 11th day of growth in contrasting temperatures (Fig. 2, B2). In lower axillary buds, the outgrowth was more at normal temperature (Fig. 2, A3) as compared to high temperature (Fig. 2, B3).

**Fig. 3:**
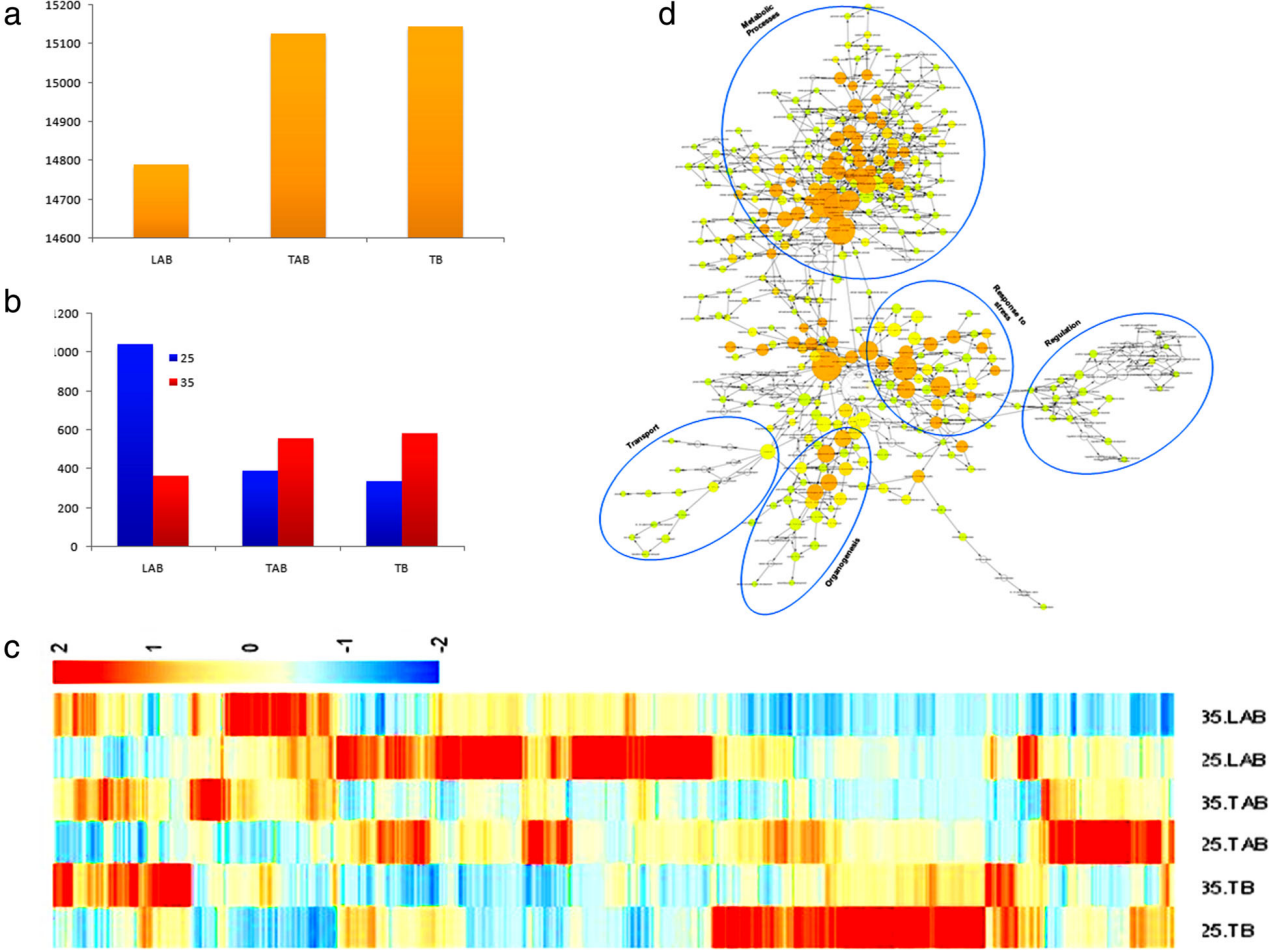
Preferential expression of genes during bud outgrowth at 25 °C and 35 °C. **a** Bar graph depicting the number of commonly expressed genes. **b** Bar graph showing the preferentially expressed genes at 25 °C and 35 °C. **c** Heatmap showing the preferentially expressed gene expression at 25 °C and 35 °C for each bud position. **d** Gene ontology (GO) enrichment (biological process) at 25 °C and 35 °C for each bud position

Considering the microscopic examination of leaves (Fig. 2c, d), the leaf mesophyll cells were more arranged and shaped in normal temperature leaves (Fig. 2; C1-C3) as compared to high temperature (Fig. 2; D1-D3), where a mild disruption was seen in the cortex. However, the influence was more prominent in top axillary leaves (Fig. 2; C1, D1), whereby high temperature caused more disrupted growth (Fig. 2, D1) than that of normal temperature (Fig. 2, C1). While observing the stomatal density, the outer surface was also seen via nail polish (Fig. 2e, f). Cell surfaces were presenting some sort of variations against normal (Fig. 2; E1-E3) and high (Fig. 2; F1-F3) temperature for TBL (E1, F1), MBL (E2-F2) and LBL (E3-F3). Figure 3(g-h) shows the transmission electron micrographs of top axillary leaves for understanding the chloroplast distribution within the cell as influenced by temperature variations.

### Temperature causes differential bud outgrowth and sugar distribution along bud positions

Significant difference (*p* < 0.01) of sucrose accumulation was noted at lower axillary buds in normal temperature as compared to high temperature (Fig. 1). However, the difference was not significant at top buds and top axillary buds at both the temperatures except that the sucrose levels were slightly high in high temperature. Significant effect of temperature can be seen in bud length at both temperature regimes, whereby high temperature suppressed the bud outgrowth (Fig. 1). Internodal distance was higher in normal temperature plants as compared to high temperature.

### Transcriptome analysis of buds at different positions

The transcriptome analysis of buds from different positions can give important system-level insights into the molecular regulatory mechanism behind bud initiation and outgrowth. A total of 13 billion high-quality reads (average ~ 65 million reads from each sample) were generated for all bud samples and mapped to chrysanthemum genome (v2.0) using TopHat. The mapped reads were processed via cuffdiff to generate normalized expression as FPKM for each transcript. The number of expressed genes varied from 60 to 70% in different tissues. About 10–15% of genes showed high expression (FPKM ≥10) (Supplementary Table 1). Overall, these analyses exhibited enough coverage of transcriptome during bud outgrowth in chrysanthemum.

### Transcriptomic comparison revealed dynamic relationships among bud stages

To know the transcriptomic differences in bud outgrowth at two different temperatures, we performed principal component analysis (PCA) and hierarchical clustering based on spearman correlation coefficient analysis of average FPKM values of all the genes expressed in at least one of the 3 tissue samples (Supplementary Fig. 2).

The tissues exhibiting high correlation are supposed to have similarity in transcriptomes and activities. These analyses pointed out higher correlation among similar bud developmental stages between two temperature regimes. It can be seen that bud transcriptomes in 35 °C were clustering differently as compared to 25 °C (Supplementary Fig. 2a). 35 °C TB and LAB were clustering more close than any of the other tissues and these two were closely placed with 25 °C TB, suggesting some point of coordination between the two temperature regimes (Supplementary Fig. 2b).

### Preferential gene expression during bud outgrowth

Stage specificity (SS) algorithm was applied, with SS score greater than or equal to 0.05, to point out the genes expressed commonly and specifically at a particular bud position in both the temperatures. Due to huge number of data, only those genes were selected with FPKM ≥5. A total of 15,144, 15,127 and 14,791 genes were found to be common in TB, TAB and LAB, respectively, at both the temperatures (Fig. 3a). However, 339 genes were expressing specifically in TB at 25 °C and 581 at 35 °C; 391 and 559 were specific to TAB at 25 °C and 35 °C, respectively; 1043 and 336 were defined as specific to LAB at 25 °C and 35 °C, respectively (Fig. 3b).

A heatmap showing the stage-specific expression of genes in chrysanthemum axillary buds is shown in Fig. 3c. The analysis of gene ontology (GO) enrichment of all the specifically expressed genes at two different temperatures exhibited the genes mainly related to metabolic processes, response to stress, growth regulation, transport, organogenesis, cell cycle, cell division and hormonal regulation (Fig. 3d). These processes are well established as integral regulators of bud growth and development.

### Differentially expressed gene sets between 25 °C and 35 °C at different bud positions

**Fig. 4:**
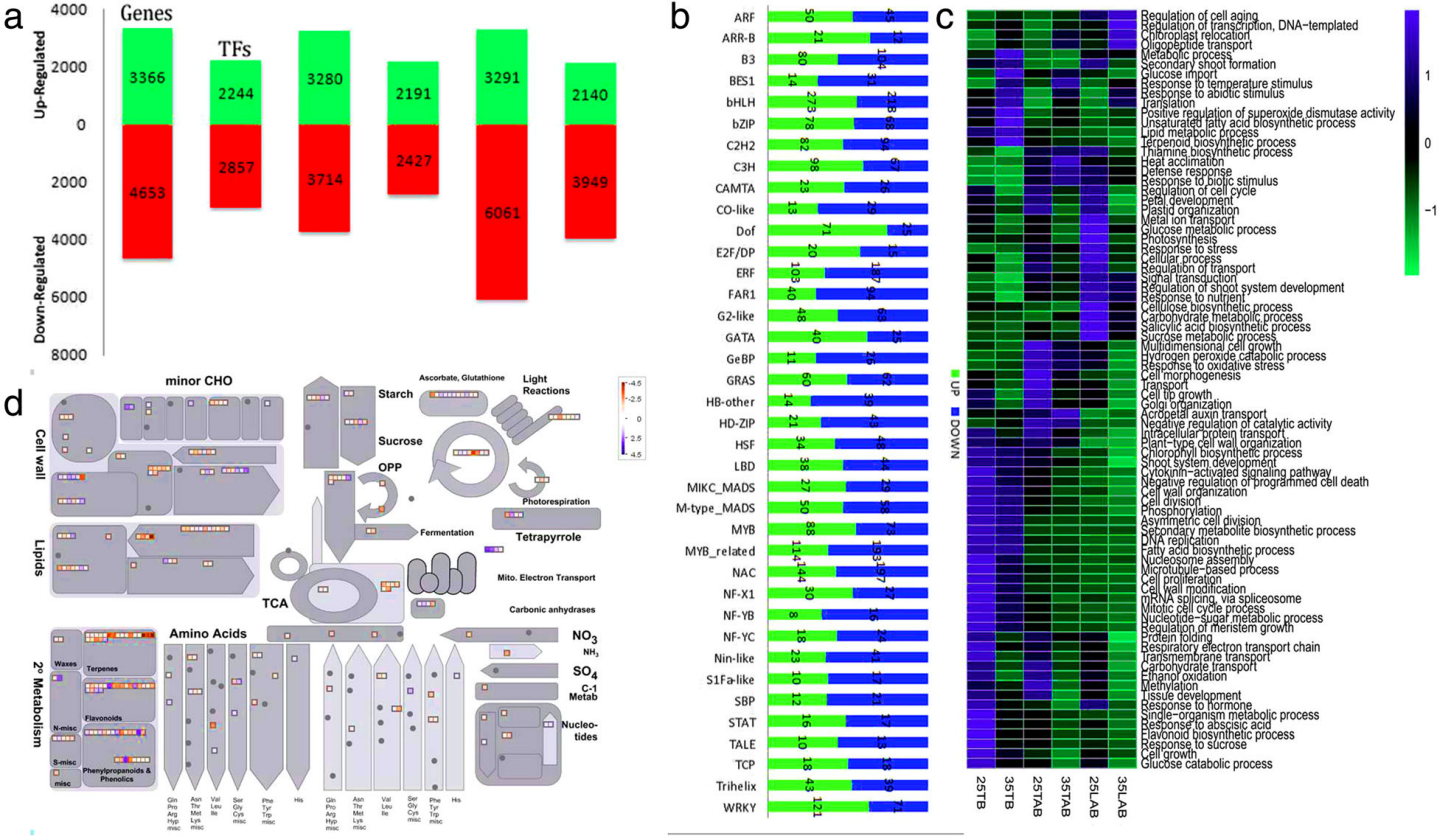
DEGs at 25 °C and 35 °C. **a** The number of up-regulated and down-regulated DEGs at each bud position, including genes and transcriptional factors. **b** The number of genes representing different transcriptional families in TAB. **c** Gene ontology enrichment (biological process), showing up- and down-regulated genes at different temperatures for TB, TAB and LAB. **d** Metabolic pathways in association with differential expression in TAB at different temperatures. Blue color represents higher expression and the red color shows lower expression

A total of 3366, 3280 and 3291 genes were up-regulated in TB, TAB and LAB, respectively; while, 4653, 3714 and 6061 genes were down-regulated in TB, TAB and LAB, respectively. A significant number of transcription factors were also detected as differentially expressing, including 2244, 2191 and 2140 up-regulated TFs in TB, TAB and LAB, respectively; and 2857, 2427 and 3949 down-regulated TFs in TB, TAB and LAB, respectively (Fig. 4a). Top axillary position offers the nascent buds appearing under different temperatures; therefore, focusing on the TABs can give a better idea about the effect of high temperature. TFs were specifically analyzed for this stage (Fig. 4b). Plenty of TF families exhibited differential expression in TAB, suggesting diverse functions during bud outgrowth (Fig. 4b). Major TF families include, ARF, B3, ERF, GRAS, MIKC_MADS, MYB, NAC, WRK and TCP. Presence of these families point out the involvement of divers activities including, cell differentiation (ARF), hormonal signalling pathways (ARF), cytokinin signalling (ARR-B). These families showed up-regulation in 35 °C as compared to 25 °C. WRK, the important transcriptional factor family for stress responses, showed significant up-regulation in response to high temperature in TAB.

The GO enrichment analysis of DEGs in different bud positions pointed out a number of biological processes uniquely overrepresented at TB, TAB and LAB. Different terms related to cell division, cell cycle and cell growth were significantly enriched in the genes with elevated expressions at TAB. Similarly, GO terms associated with cellular components were also showing high expression at Tab. A wide range of GO terms were evident at all stages of bud outgrowth, including organogenesis, DNA replication, phosphorylation, hormonal responses, sucrose metabolism, transport, regulation of shoot development, photosynthesis etc. (Fig. 4c).

To ascertain the metabolic pathways responsible for bud outgrowth at TAB, the expression profiles of DEGs were overlaid onto the already known metabolic pathways using MapMan tool (Fig. 4d). Differential activity was observed about certain metabolic pathways at TAB under contrasting temperatures. Considerable differences were seen, under both the temperatures, in the activity of the genes responsible for starch biosynthesis, especially the sucrose metabolism. Moreover, hormonal pathways were also evident in the temperature responses in TAB, including the signalling for auxin, cytokinins and abscisic acid. The genes governing the starch metabolism and photosynthesis were also active in TAB at high temperature (Fig. 4d). A significant proportion of cell wall related genes expressed at higher levels in normal temperature top axillary buds, suggesting higher cell activity at suitable ambient environment than that of higher temperature, where temperature fluctuations beyond a normal range can hinder a range of cell activities.

### Hormonal networks are also involved in temperature sensing for bud kinetics

**Fig. 5:**
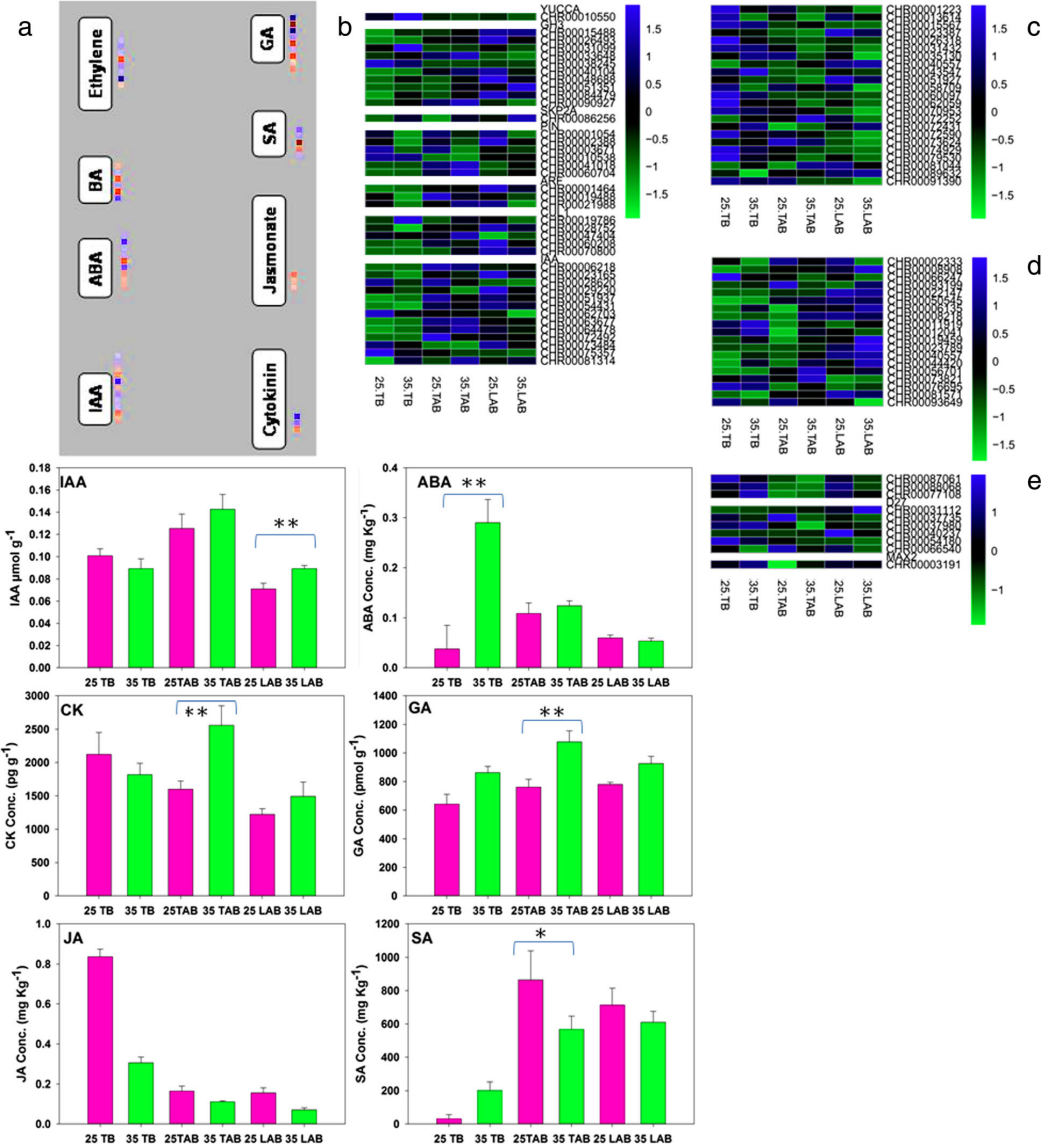
Hormonal concentrations [IAA (indole acetic acid), ABA (abscisic acid), CK (cytokinins), GA (gibberellic acid), JA (jasmonic acid) and Sa (salicylic acid)], and DEGs related to hormonal control of buds, including top buds, top axillary buds and lower axillary buds. **a** Mapman based identification of DEG groups involving different hormones, including auxins, cytokinins, abscisic acid (ABA). **b** Gene expression values (FPKM based) for auxin related genes at 25 °C and 35 °C. **c** Gene expression values (FPKM based) for cytokinins related genes at 25 °C and 35 °C. **d** Gene expression values (FPKM based) for ABA related genes at 25 °C and 35 °C. **e** Gene expression values (FPKM based) for strigolactone related genes at 25 °C and 35 °C. Data are shown as mean ± SE of three biological replicates. Asterisks on specific terms show significant differences between the treatment conditions 25 °C and 35 °C for each bud position at *p* < 0.05(*) and *p* < 0.01(**) level

A number of genes were identified involving in auxin biosynthesis and signalling with significant different set of expressions at 25 °C and 35 °C (Fig. 5b). Some of the candidate genes already known for auxin control have also been mined through sequencing data, including PIN, IAA, YUCCA, SKP2A and CUL1. Most of the genes are showing high expression in 25 °C as compared to 35 °C. Similar results can be seen in the case of cytokinins wherein most of the genes exhibited high expression values in normal temperature regime as compared to high temperature (Fig. 5c).

The transcriptome analysis also depicted a role played by these hormones in axillary buds at two different temperature regimes (Fig. 5a). However, the role of strigolactone and ABA related genes was different from those of auxin and cytokinins related genes (Fig. 5d). ABA related genes exhibited quite high expression (a measure of FPKM value) in high temperature samples as compared to normal temperature. Fluctuations at TAB for IAA and CK at high temperature are obvious as compared to normal temperature (Fig. 5; IAA, CK). However, the expression intensity and difference was prominent in top axillary buds which are the most probable sites for receiving high temperature influence. Significantly high expression intensities of ABA related genes are obvious at top axillary buds, showing that high temperature reception was negatively expressed at top axillary sites to restrict bud outgrowth. However, the differences are considerable at other bud positions, including top buds and lower axillary buds. High ABA concentration at TB and TAB at high temperature suggests thermal regulation of bud through ABA (Fig. 5ABA).

Some genes were found related to strigolactone (Fig. 5e). The difference was not very significant between two temperatures. However, relatively high gene expression was seen in two candidate genes (D27 and MAX2) at 35 °C as compared to 25 °C.

### Identification of coexpressed gene modules for selected morphological leaf and bud traits

**Fig. 6:**
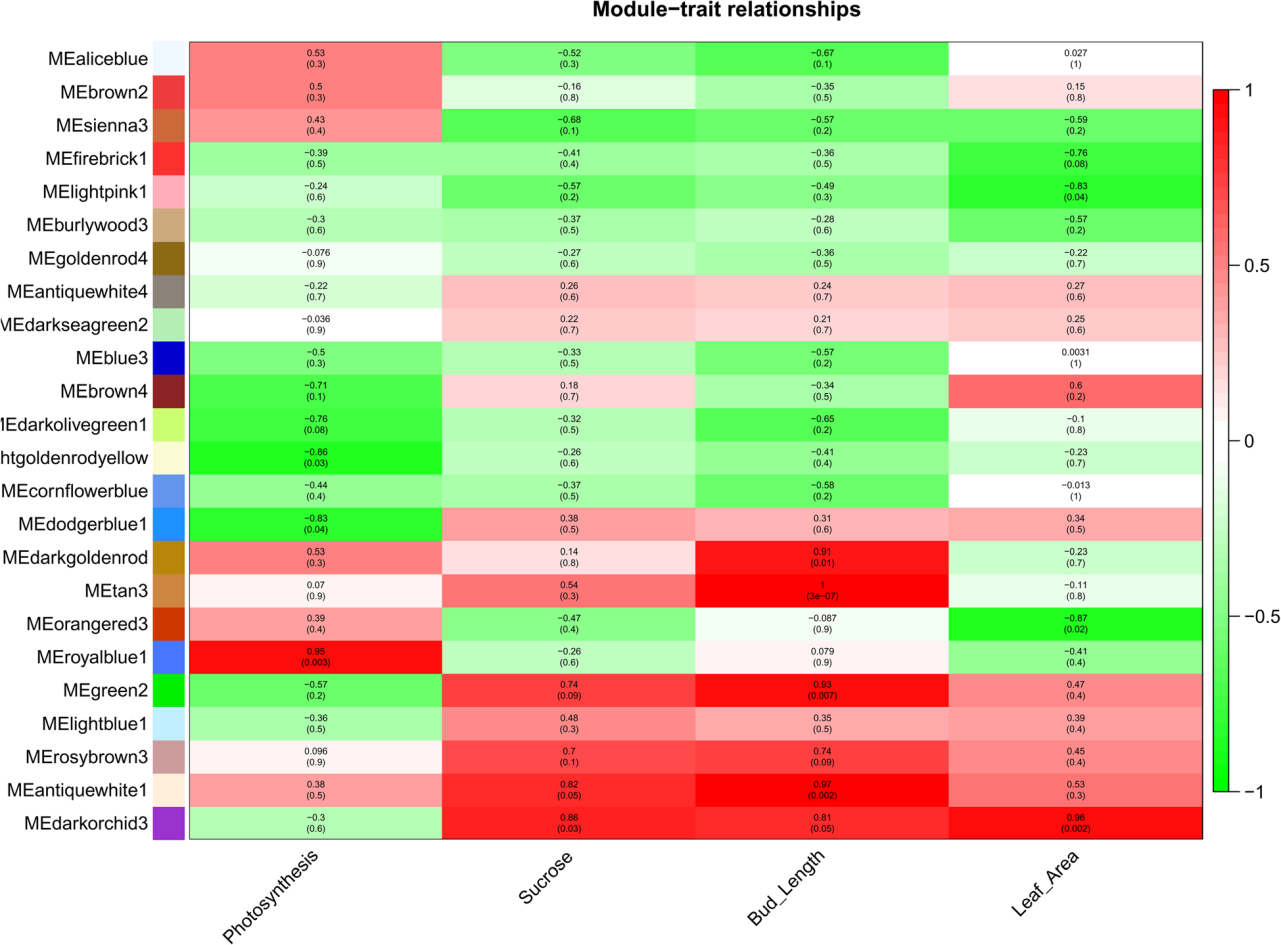
Coexpression network analysis during axillary bud outgrowth in association with leaf (leaf area, photosynthesis) and bud (sucrose, bud length) attributes

To understand the gene regulatory networks during bud outgrowth, we performed weighted gene co-expression network analysis (WGCNA) in association with leaf characteristics (leaf area and photosynthetic rate) and bud characteristics (bud length and sucrose concentration) (Fig. 6). The genes showing a FPKM ≥1 were considered for this analysis due to large amount of data. A total of 24 modules were observed for bud outgrowth at contrasting temperatures (Fig. 6). Among these modules, MEdarkorchid3 was the most prominent in representing highly upregulated genes involved in leaf area and bud-sucrose and length manipulations. Some modules exhibited strong responses for bud length, including MEdarkorchid3, MEantiquewhite1, MEgreen2, MEroyalblue 1, MEtan3 and MEdarkgoldenrod (Fig. 6). However, the rate of photosynthesis and the leaf area were only prominent in MEroyalblue 1 and MEdarkorchid3, respectively. In the case of sucrose, high expression modules included MEdarkorchid3, MEantiquewhite1, MErosybrown3 and MEgreen2, suggesting the involvement of sugar homeostasis in bud kinetics.

Hub genes were identified using Cytoscape for MEantiquewhite1 (trpC and GRR1) (supplementary Fig. 3) and MEGreen2 (UBC12 and CYP17) (supplementary Fig. 4). All bud positions showed difference for these hub genes at different temperatures. However, the significant differences can be seen at lower axillary buds (supplementary Fig. 5). The trpC plays role in cell wall synthesis and the GRR1 works in auxin biosynthetic pathway. UBC12 acts as an enzyme in auxin biosynthetic pathways and the CYP17 plays a role in steroid hormone biosynthesis.

## Discussion

The molecular regulation for the axillary bud outgrowth is poorly defined in chrysanthemum while the success of cut chrysanthemum solely depends on the vigor of single stalk devoid of axillary branches. However, so far, the efforts to nullify branching have not yet been successful due to unavailability of a profound regulatory network to knock out. We used leaf morpho-physiological indicators and RNA-seq approach from axillary buds to ascertain the transcriptome dynamics at three bud positions influenced by contrasting temperature regimes. A considerable proportion of chrysanthemum genes were shown to be expressed in at least one of the bud positions. High throughput RNA-Seq facilitated the mining of new genes with differential expression profiles. The expression information around the three bud positions exhibited significant reproducibility at two temperatures and each bud position was distinguished from the other in the principal component analysis, suggesting prominent gene expression changes among bud stages. The comprehensive analysis of transcriptome data along with coexpression networks pointed out a number of specific and coregulated transcriptional plans associated with different bud outgrowth stages. Moreover, sucrose, hormonal contents and the morpho-physiological indicators from leaf also strengthened the deep impact of temperature on the axillary bud sites.

In the temperate zone, the bud burst is caused mainly by temperature [37, 38]. The tillering extent can affect leaf area, plant density and the light interception [2]. In our study, leaf area was narrowed down by high temperature (Fig. 1). Bud development starts with the initiation of meristem [12] and then at a certain transition stage, the outgrowth fate is driven by intrinsic and extrinsic factors [39, 40]. A plant may be unable to develop an axillary growth due to malformation of meristem or outgrowth inhibition of axillary buds [41].

Environmental fluctuations may trigger differential expression of certain miRNAs to compete with stress conditions [42]. Some of miRNAs have been identified in plants related to stress, including drought [43], nutrient deficiency [44], heat [45] and cold [46]. Most of the plants can adjust their biochemical and physiological processes by fluctuating proline contents, MDA, hydrogen peroxide and sucrose contents to manage temperature variations [47, 48]. Interesting fluctuations were observed in ROS and antioxidant species in leaves at 35 °C as compared to 25 °C. However, most of them showed high values in lower bud leaves as compared to top bud leaves in normal temperature. The chloroplast is a potent sensor for stress responses and environmental changes [49]. Slight movement of chloroplast can be seen in leaves under 35 °C (Fig. 2g, h).

Temperature elevation beyond a certain limit negatively affects plant growth by delaying growth and several gene units can be involved [42]. For example, temperature stress can disturb the nutrient balance, hormonal and metabolic homeostasis in plants [50, 51], thereby impacting the regulatory machinery behind bud outgrowth. Temperature change modifies the hormonal levels inside the plant body [52]. ARFs are the transcriptional factors that control the expression of genes induced by auxin through their binding to ARPs (auxin-responsive promoters) [53].

Researchers have shown that bud outgrowth status is controlled by numerous factors, including mainly the growth promoters such as sugars, cytokinins and inhibitors like auxin, ABA and strigolactone [11, 12, 54–58]. Relatively high amounts of ABA at top buds and top axillary buds suggest high temperature control of axillary buds by ABA (Fig. 5). However, higher IAA concentrations at TAB and LAB at 35 °C point more movement of auxin from top buds towards lower bud positions as compared to normal temperature (Fig. 5). Through decades of research, a group of genes expressing in sorghum buds (i.e., *GT1/BRC1/TB1/MAX2*) has been identified to repress bud outgrowth until environmental cues, cytokinins and sugar signaling are permissible for axillary bud outgrowth [2]. Strigolactones are the key controllers of axillary shoots that unite to DAD2/MAX2:D14 complex and work through *GT1/BRCI/TB1* pathway [12]. *MAX2* inhibits bud outgrowth through encoding an F-box protein [13, 14]. *RwMAX*, from rose, was downregulated by sucrose supplies [11]. In stem, the level of cytokinins is degraded by auxin-induced downregulation of cytokinins biosynthesis gene, whereas abscisic acid (ABA) expresses an auxin-independent inhibition of bud outgrowth [17, 54, 59]. In our study, high expression of *MAX2* at TAB at 35 °C shows strigolactone-mediated inhibition of axillary sites by high temperature (Fig. 5e).

The differential increase in bud length at different positions suggested the differential role of two temperatures in cell division. The gene ontology enrichment analysis shows high rate (higher FPKM) of cell cycle and cell division in normal temperature especially at TAB. The extended mitosis can be seen at normal temperature. High mitotic activity causes higher gluconeogenesis, resulting in increased seed size [60]. We noted considerable transcriptional activity of genes for cell growth, cell cycle, heat stress and GO enrichment, showing significant differences at both the temperatures (Fig. 4). Moreover, several genes related to cell expansion, storage compounds and fatty acid biosynthesis shown higher transcriptional activity for normal temperature (Fig. 4c). A number of TF families are studied to be involved in organ development [61–63]; however, a few of them are considered to be involved in temperature-induced bud outgrowth. Our study found plenty of TFs to be involved in differential bud outgrowth at contrasting temperatures, especially at TABs. Some known TF families were shown among the differentially expressing TFs; however, the exact function is still unclear for most of these genes. Some known TF families, for example NAC, ARF and WRKY, which expressed differently at two temperature regimes, are well established in their role in organ development [7, 61–64]. Differential expression intensities of same family members at different bud positions and different temperatures may involve different regulatory pathways, thereby determining position-specific bud development. To get better understanding of this, we used coexpression network analysis to mine unique and common gene groups associated with bud outgrowth at contrasting temperatures.

Ascertaining the transcription modules can disclose gene regulatory networks governing biological processes linked with bud and seed development [2, 7, 42, 65]. Therefore, we created transcription modules (by connecting transcriptional factors with their respective binding motifs and coexpressed target genes) for top axillary buds which are considered to be the crucial bud outgrowth stage to study temperature influence. Although an extensive overlap was observed for TAB at 25 °C and 35 °C, there were several components pertinent to a specific transcriptional accumulation, suggesting uniqueness of the transcription modules for each temperature regime. The modules with opposite expression genes were mainly concerned with cell cycle, growth, cell size, histone modification and energy. Several components of these modules have previously been implicated in different aspects of bud and seed development [2, 65–70]. Thus, our study demonstrated that transcriptional module construction along with coexpression networks can do a great deal to comprehend the inherent mechanism governing agronomic traits of bud outgrowth. However, further studies about each network member are needed to elucidate the whole diagram of GRNs.

## Conclusion

The regulation of axillary bud by environmental and hormonal signals is well established; however, their inherent mechanisms are largely unknown in chrysanthemum. Studying high temperature as a vital factor inhibiting but outgrowth provides a much better understanding of how stooling is controlled during plant growth. In the present study, RNA-Seq data coupled with morpho-physiological integrators from three bud positions at two temperature regimes brings a robust source to understand bud outgrowth status influenced by high temperature in cut chrysanthemum. Our results provided the evidence that different bud positions exhibited relative susceptibility towards temperature changes, especially the top axillary buds showed distinguished response for high temperature in chrysanthemum. Our results showed that photosynthetic leaf area, physiological indicators, hormonal fluctuations and sucrose utilization are significantly changed, indicating that they were involved in inhibiting the axillary bud outgrowth by high temperature. Transcriptomic comparison revealed amount of bud position-specific expression gene sets. Using WGCNA, we identified important modules highly associated with morphological leaf and bud traits. Our results provide helpful information for elucidating the regulatory mechanism of temperature on axillary bud growth in chrysanthemum.

## Methods

### Plant material and growth conditions

Cuttings of the *Chrysanthemum morifolium* variety ‘Jinba’ were obtained from the Chrysanthemum Germplasm Resource Preserving Nursery (Beijing Forestry University, Beijing, China). Cuttings of uniform length, containing at least two buds were obtained from mother plant and were grown in 50H-Cutting tray Drip trays in the greenhouse of Beijing Forestry University. After 20 days, the seedlings were grown into pots. Two month old seedlings at 15 axillary bud stage were transferred to controlled temperature chambers fitted with uniform light (Philips T8 TLD36/33 cold white tube, 120 μmol m^− 2^ s^− 1^ optical density). One of the chambers was set to day and night temperature of 35/25 °C, respectively, and was regarded as high temperature regime. The other chamber was set to 25/15 °C and was regarded as normal temperature regime. Both the chambers were provided with equal light intensities with a day to night duration of 16/8 h. A total of 45 plants were kept in each of the high and normal temperature chambers.

### Sampling procedure

After 11 days of growth in contrasting temperatures, the sampling was performed from top buds (TB), top axillary buds (TAB) and lower axillary buds (LAB). The sampling portion was the rectangular stem portion including the axillary bud (Fig. 1). The leaves around the top bud were regarded as the top bud leaves (TBL) and those around top and lower axillary buds as middle bud leaves (MBL) and lower bud leaves (LBL), respectively. Leaf samples were also collected along with bud samples for physiological indices. Single repeat included sampling from 15 plants, containing 15 TBs, 45 TABs and 45 LABs. Thus, three repeats were collected from 45 plants in each temperature regime. Sampling was done in three biological repeats. Samples were collected in liquid nitrogen and stored at − 80 °C until RNA extraction or physiological analyses.

### Morphological parameters

#### Bud length

Bud length (mm) was measured every week using digital Vernier calipers.

#### Leaf area (cm^2^)

After 11 days of growth under contrasting temperatures, top leaf, top axillary leaf and lower axillary leaf were scanned along with proper scale. Scanned pictures were used to measure leaf area by ImageJ software [71].

#### Wet to dry mass ratio

Top bud leaf (TL), middle bud leaf (MBL) and the lower bud leaf (LBL) were selected from 11 days old plants and weighted initially to note the wet weights. The leaves were packed in paper and kept overnight in an incubator set to 65 °C. Crack-dried leaves were weighted again to take the dry weights. Wet to dry ratios were calculated by dividing the wet weight of each leaf to its dry weight.

#### Gas exchange and photosynthetic pigments

Net rate of photosynthesis (Pn), water conductance (Cw), intercellular CO_2_ concentration (Ci) and transpiration rate (E) were measured using a portable measuring system (Ecotek, China). The environmental parameters were: leaf temperature 25 °C, relative air humidity 80%, 1200 μmol m^− 2^ s^− 1^ photosynthetic photon flux density (PPFD) and 400 ± 5 μmol mol^− 1^ of ambient CO_2_ concentration.

Chlorophyll pigments were measured using the method of Zhang [72] with little modification. Briefly, 1 g of leaf sample was homogenized in 80% (v/v) acetone solution, followed by centrifugation at 10,000 g for 10 min at 4 °C. The supernatant was collected to measure absorbance at 663, 645 and 470 nm to measure chlorophyll a (Chl a), chlorophyll b (Chl b), carotenoids (Caro), and total chlorophyll (Chl) considering the calculations by Lichtenthaler [73].

#### Physiological indices

Protein, Malondialdehyde contents and the antioxidant enzyme activities were measured following the protocol by Chen and Zhang [74].

#### Stomatal density

Stomatal density was measured following the procedure described by Hopper et al. [71]. Stomatal density was ascertained using ImajeJ (National Institutes of Health, Bethesda, MD, USA) applying the plug-in of cell counter [75].

### Microscopic documentation of axillary buds under different temperature regimes

#### Paraffin sectioning

Stem cuttings containing single axillary buds were excised to see bud activities at micro level. After every 24 h the buds were fixed in FAA (formalin-acetic acid-alcohol) containing 70% ethanol, 37% formaldehyde acetic acid at a ratio of 18:1:1. Buds were then dehydrated using butyl alcohol series and embedded in paraffin. Embedded samples were cut into 10 μm thick strips using rotary microtone and then placed on microscopic slides. Slides were kept overnight at 40 °C and stained in Safranin-O and fast-green staining series (Kebrom and Mullet, 2015) and were mounted using few drops of Permount medium (Fisher Scientific, Waltham, MA, USA). The slides were covered with cover glass and observed using a bright-field microscope.

#### Transmission electron microscopy

Top axillary leaf mesophyll cells were anatomically analyzed using transmission electron microscopy [76, 77]. 2 mm^2^ leaf sections were cut parallel to the midrib and immersed in 2.5% (v/v) glutaraldehyde solution. The solution was then replaced with fresh fixative. After proper washing, the samples were fixed in 1% OsO_4_ (w/v) with K_3_Fe(CN)_6_ in 0.1 M Sodium carbohydrate buffer. Successive ethanol series were run to dry the samples (including staining with 2% uranyl acetate at 50% ethanol step), followed by embedding in Spurr’s resin. Extremely thin sections were cut using an ultramicrotome (Leica EM UC6). These sections were further stained with uranyl acetate and lead citrate. Sections were finally viewed with a high definition transmission electron microscope (Tecnai 12, Philips, The Netherlands).

#### Measurement of sucrose concentration

The concentration of sucrose was estimated following the methodology of Yuan et al. [78] with slight modification. In short, buds were finely ground in liquid nitrogen and extracted three times (at 80 °C) in 80% ethyl alcohol. The pooled supernatants were filtered using carbon black, making a final volume of 25 ml by adding distilled water into filtrate. The reaction mixture contained 100 μl of 2 N NaOH and 900 μl of extract. This solution was boiled at 99 °C in a water bath for 10 min, followed by cooling for 5 min. After that, 1 ml of 0.1% resorcinol and 3 ml of 10 N HCl were added to this reaction mixture and heated at 80 °C for 1 h. Absorbance was observed at 480 nm using a UV-1700 PharmaSpec spectrophotometer (Shimadzu Corporation, Kyoto, Japan). Sucrose solution of 20 μg/ml was used to obtain a standard curve at a proper correlation coefficient (R^2^ = 0.998).

### Analysis of hormones

Major hormones related to bud outgrowth were analysed with HPLC-MS/MS (Aglient) as described by Pan et al. [79].

### RNA-seq library preparation and sequencing

Total RNA was extracted from frozen buds using MiniBESTplant RNA extraction kit (TaKaRa) including DNA removal as well, finally obtaining DNA-free RNA. All the 18 libraries (6 samples in three biological replicates) were sequenced on Illumina platform (HiSeq 2000) to produce paired-end sequence reads of 150-nucleotide long. The raw data were analyzed to understand various parameters and the high-quality reads were sorted with NGS QC Toolkit (v2.3). The quality reads were mapped on the *Chrysanthemum nankingense* genome [1] using TopHat (v2.0.0) with default parameters. FPKM (fragments per kilobase of transcript length per million mapped reads) values were obtained for all the genes in each sample by processing the mapped data through Cufflinks (v2.0.2). Spearman correlation coefficient (SCC) was applied to determine the correlation between the biological repeats. Principal component analysis (PCA) and hierarchical clustering were executed using prcomp and corrplot utilities of R package [7]. Differential expression was ascertained between the samples using Cuffdiff and the genes showing at least twofold difference in expression with a corrected *P*-value (*q*-value) of < 0.05 after the adjustment of false discovery rate. Stage specificity (SS) scoring algorithm was used to identify preferentially expressed/stage-specific genes. SS scoring algorithm identifies stage-specific gene by comparing the expression of that genes in a specific stage with its maximum expression value in other stages as explained in previous researches [7, 80]. Higher the SS score of gene in a given stage more significant is the expression of that gene at that stage. A selected set of genes was used to generate heatmap using pheatmap and ggplot2 utilities of R.

### Gene ontology and pathway enrichment analysis

GO enrichment analysis was performed for DEGs using BINGO plug-in of Cytoscape [7, 81]. For each GO term, the *P*-value was calculate and corrected using the error correction method by Benjamini Hoschberg [7]. The GO terms with *q*-value of ≤0.05 were regarded as significantly enriched. MapMan (v3.6.0RC1) was used to generate pathway enrichment analysis of different gene sets with the best Arabidopsis (TAIR10) homolog.

### Weighted gene coexpression network analysis

For the construction of coexpression modules, the WGCAN packages were used [82, 83]. Using log_2_ (1 + FPKM) values, a matrix containing pairwise SCCs between all gene pairs was produced, followed by transformation into an adjacency matrix using the following formula: adjacency value = |(1 + correlation)/2|^β^. β shows soft threshold value for the correlation matrix, giving elevated weight to the strongest correlations while reserving inter-gene connectivity. A β magnitude of 12 was chosen on the basis of scale-free criterion for topology described previously [7, 82]. Adjacency matrix, thus obtained, was changed to a TO (topology overlap) matrix through TOM similarity algorithm, followed by hierarchical clustering of genes on the basis of TO similarity. Hierarchical clustering dendrogram was cut via dynamic tree-cutting algorithm and the modules were defined by combining the branches to a stable number of clusters [83]. A summary profile called module eigengene (ME) was calculated for each module using PCA. Those modules were retained with higher TO value as compared to the TO values of randomly selected gene modules. GO enrichment analysis was performed for each module.

### Statistical analysis

The data was analysed using One-way ANOVA in SPSS software (SPSS Inc., Chicago, IL, USA; ver. 16.0). Significant variations are indicated at *p < 0.05* or *p* < *0.01* level.

## Supplementary information

**Additional file 1: Figure S1.** Bud length and bud morphology at different plant heights. **Figure S2.** Correlation between the transcriptomes of different bud positions. **Figure S3.** Candidate gene selection for Antiquewhite1. **Figure S4.** Candidate gene selection for Green2. **Figure S5.** FPKM values of selective candidate genes from ‘Antiquewhite1’ (tprC, GRR1) and ‘Green2’ (UBC12, CYP17) modules of bud length. **Table S1.** FPKM based grouping of mapped reads.

## Data Availability

The data sets are included within the article and its Additional files. The raw sequence data reported in this paper have been deposited in the NCBI under the BioProgect ID PRJNA608820. The raw sequence data are also available in the Genome Sequence Archive in BIG Data Center, Beijing Institute of Genomics (BIG), Chinese Academy of Sciences, under accession numbers CRA002314.

## Abbreviations

ABA: Abscisic acid
Caro: Carotenoids
CAT: Catalase
Chl: Total chlorophyll
Chl a: Chlorophyll a
Chl b: Chlorophyll b
Ci: Intercellular CO_2_ concentration
CK: Cytokinins
Cw: Water conductance
DEGs: Differentially expressed genes
FAA: Formalin-acetic acid-alcohol
FPKM: Fragments Per Kilobase of transcript per Million mapped reads
GO: Gene ontology
IAA: Indole-3-acetic acid
MDA: Malondialdehyde
PCA: Principal component analysis
PIN: Pin-formed
Pn: Net rate of photosynthesis
POD: Peroxidase
PPFD: Photosynthetic photon flux density
ROS: Reactive oxygen species
SOD: Superoxide dismutase
TB: Top buds
TAB: Top axillary buds
LAB: Lower axillary buds
TFs: Transcription factors
TBL: Top bud leaf
MBL: Middle bud leaf
LBL: Lower bud leaf
WGCNA: Weighted Gene Co-expression Network Analysis
