# Supplementary material for: Morpho-physiological integrators, transcriptome and coexpression network analyses signify the novel molecular signatures associated with axillary bud in chrysanthemum
Source: BMC Plant Biol. 2020 Apr 7;20:145. doi: 10.1186/s12870-020-02336-0 (PMC7140574; doi:10.1186/s12870-020-02336-0)
Supplement: Supplementary file 1 — Additional file 1: Figure S1. Bud length and bud morphology at different plant heights. Figure S2. Correlation between the transcriptomes of different bud positions. Figure S3. Candidate gene selection for Antiquewhite1. Figure S4. Candidate gene selection for Green2. Figure S5. FPKM values of selective candidate genes from ‘Antiquewhite1’ (tprC, GRR1) and ‘Green2’ (UBC12, CYP17) modules of bud length. Table S1. FPKM based grouping of mapped reads. [file 12870_2020_2336_MOESM1_ESM.docx]

**Morpho-physiological integrators, transcriptome and coexpression network analyses signify the novel molecular signatures associated with axillary bud in chrysanthemum**

Sagheer Ahmad^1^, Cunquan Yuan^1,^**^*^**, QingqingYang^1^, Yujie Yang^1^, Tangren Cheng^1^, Jia Wang^1^, HuitangPan^1^, Qixiang Zhang^1,2,^**^*^**

^1^Beijing Key Laboratory of Ornamental Plants Germplasm Innovation & Molecular Breeding, National Engineering Research Center for Floriculture, Beijing Laboratory of Urban and Rural Ecological Environment, Key Laboratory of Genetics and Breeding in Forest Trees and Ornamental Plants of Ministry of Education, School of Landscape Architecture, Beijing Forestry University, Beijing, 100083, China.

^2^Beijing Advanced Innovation Center for Tree Breeding by Molecular Design, Beijing Forestry University, Beijing 100083, China.

******Corresponding author:*

Cunquan Yuan

E-mail: yuancunquan@163.com

Phone number, +8601062336321

Fax number, +8601062336321

Qixiang Zhang

E-mail, zqxbjfu@126.com

Phone number, +86 01062338347

Fax number, +86 01062336321

**Supplementary Figure 1.** Bud length and bud morphology at different plant heights.

**
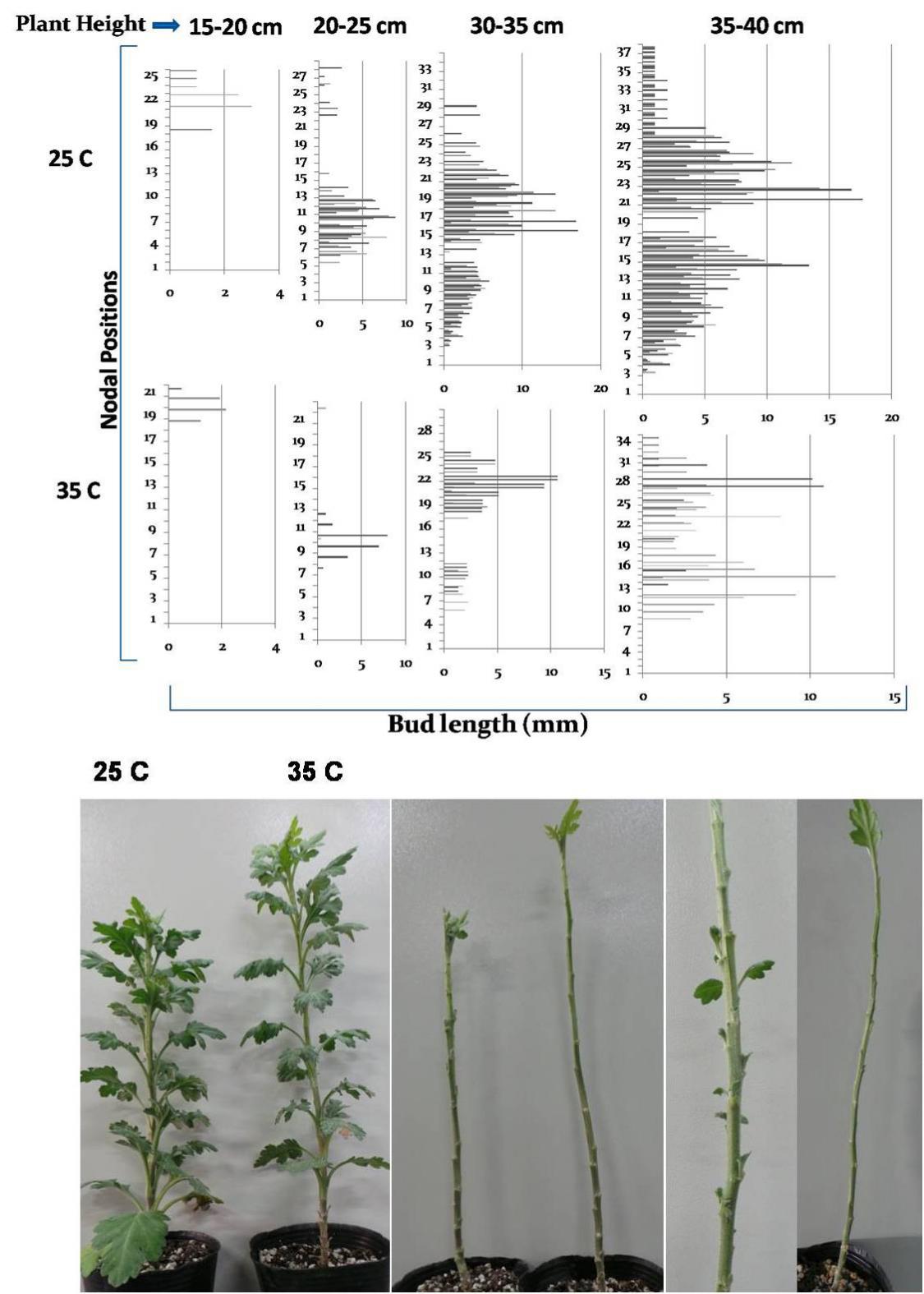
**

**Supplementary Figure 2.** Correlation between the transcriptomes of different bud positions. (Left) SCC analysis of RNA-seq data from top buds, top axillary buds and lower axillary buds at 25 ^o^C and 35 ^o^C. (Right) PCA plot exhibiting the clustering of transcriptomes of different bud outgrowth stages at 25 ^o^C and 35 ^o^C.

**
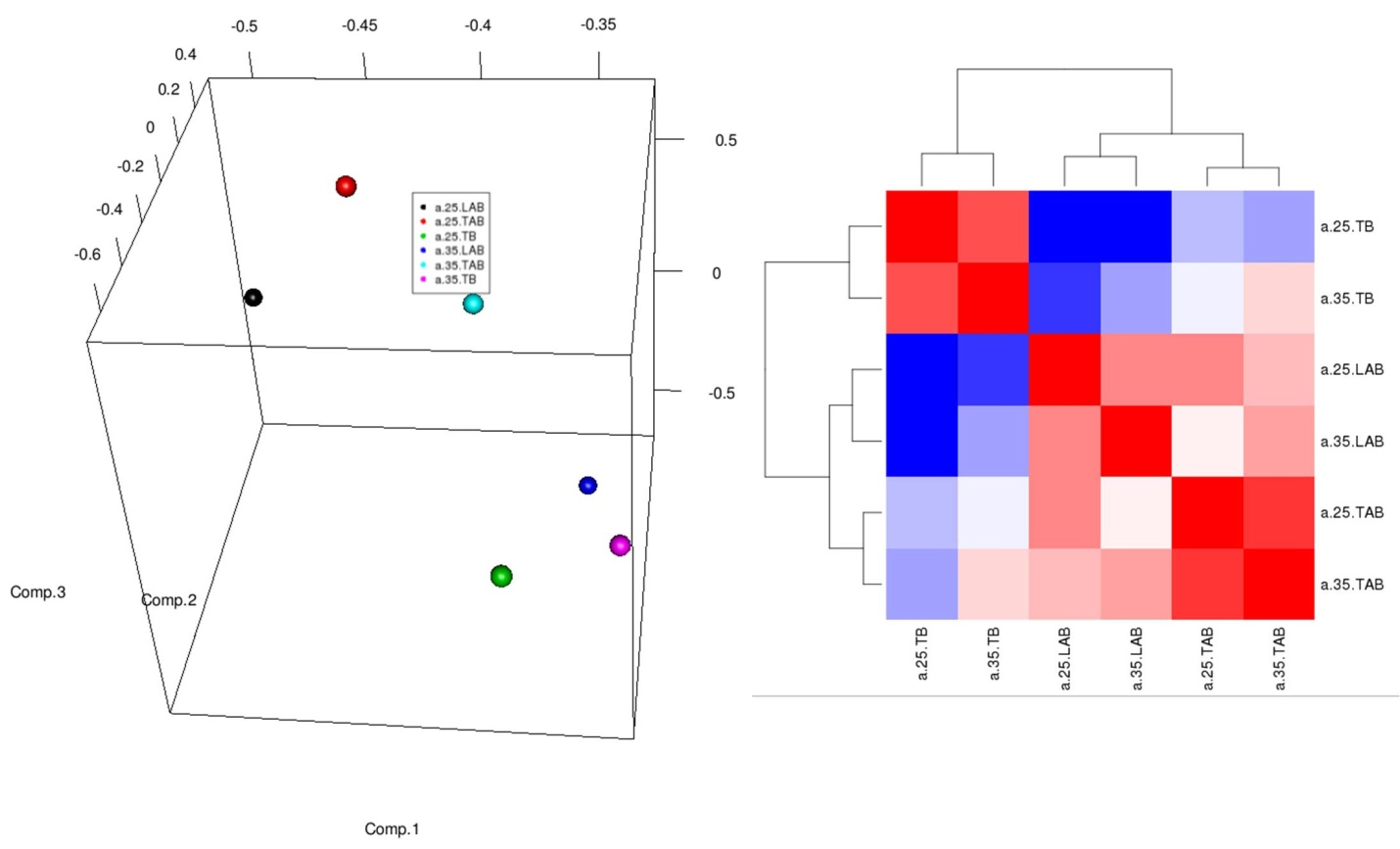
**

**Supplementary Figure 3. Candidate gene selection for Antiquewhite1**

**
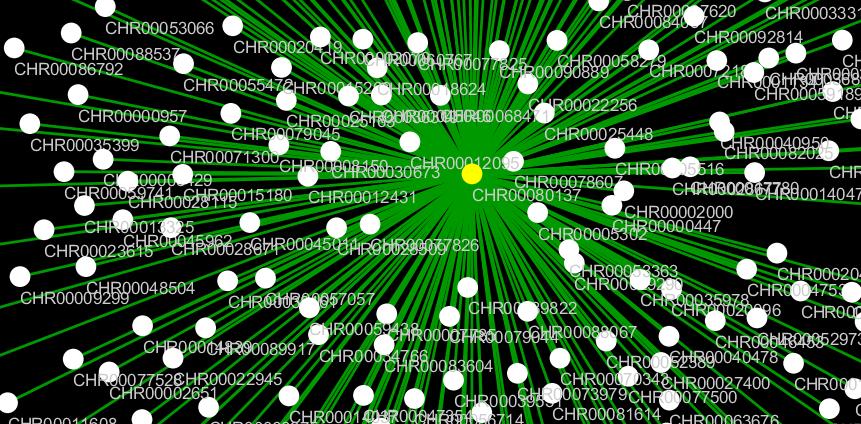
**

**Supplementary Figure 4. Candidate gene selection for Green2**

**
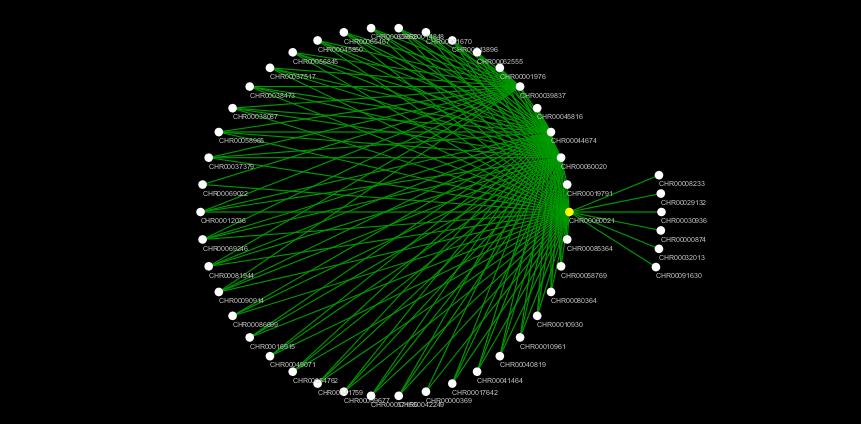
**

**Supplementary Figure 5. FPKM values of selective candidate genes from 'Antiquewhite1'**

**(tprC, GRR1) and 'Green2' (UBC12, CYP17) modules of bud length**

**
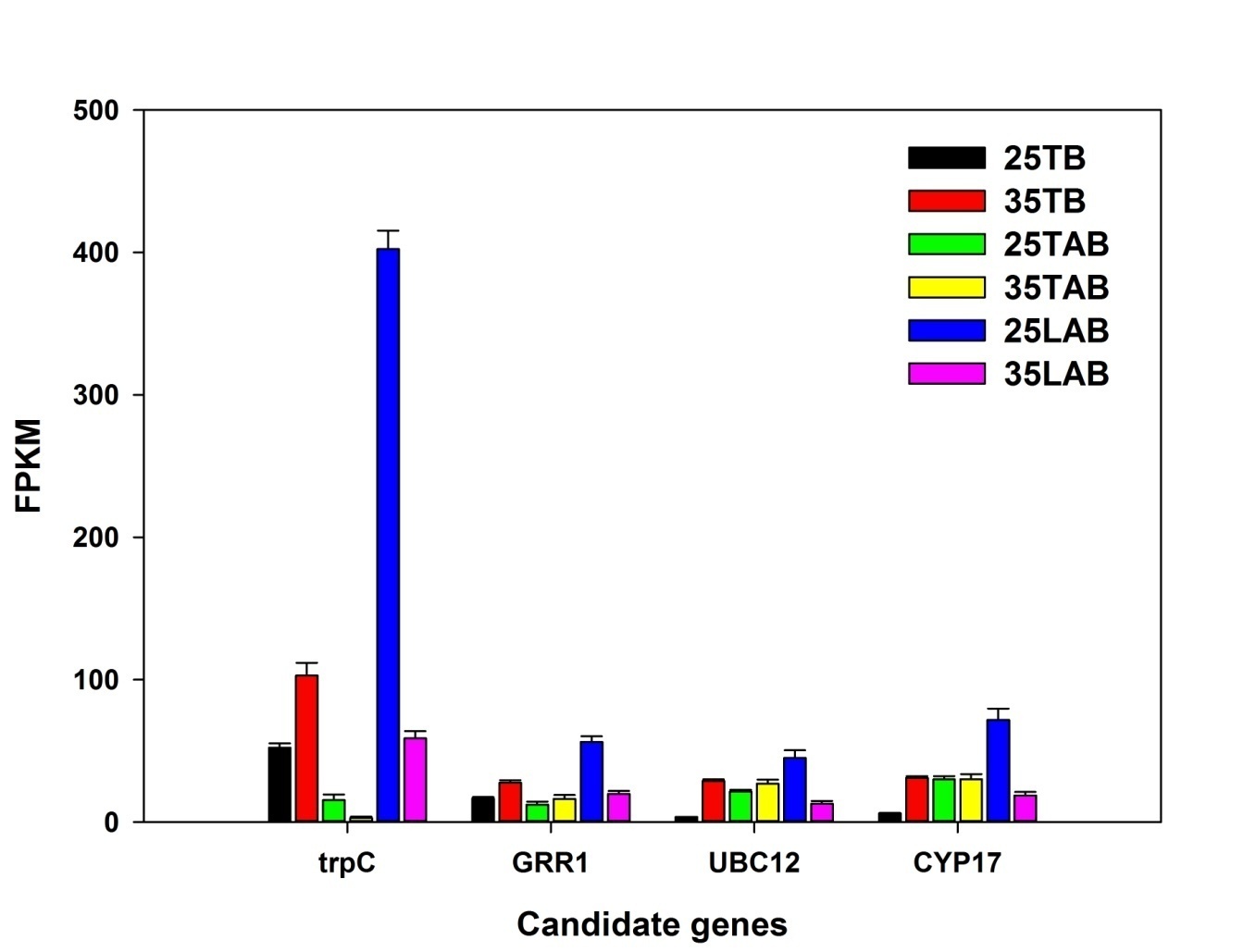
**

**Supplementary Table 1.** FPKM based grouping of mapped reads

| **FPKM** | **25 LAB** | **25 TAB** | **25 TB** | **35 LAB** | **35 TAB** | **35 TB** |
| --- | --- | --- | --- | --- | --- | --- |
| 0 | 22110 | 23421 | 22978 | 21155 | 21469 | 21845 |
| 0 -- 0.25 (> 0) | 4566 | 4335 | 5106 | 5095 | 5305 | 5301 |
| 0.25 -- 0.5 | 2334 | 2268 | 2413 | 2499 | 2435 | 2408 |
| 0.5 -- 1 | 2785 | 2714 | 2672 | 2895 | 2889 | 2897 |
| 1.0 -- 5.0 | 7847 | 7639 | 7264 | 8276 | 8011 | 7756 |
| 5.0 -- 10 | 3951 | 3713 | 3658 | 4088 | 3943 | 3873 |
| 10 -- 50 | 9056 | 8771 | 8565 | 9245 | 9065 | 8964 |
| 50 -- 100 | 2278 | 2138 | 2193 | 2042 | 2104 | 2123 |
| 100 -- 500 | 1700 | 1618 | 1690 | 1377 | 1442 | 1475 |
| 500 -- 1000 | 145 | 162 | 201 | 126 | 120 | 137 |
| > 1000 | 98 | 91 | 130 | 72 | 87 | 91 |
